# YAP1 promotes multidrug resistance of small cell lung cancer by CD74‐related signaling pathways

**DOI:** 10.1002/cam4.2668

**Published:** 2019-11-06

**Authors:** Yongchun Song, Yanqin Sun, Yingying Lei, Kui Yang, Ruixiang Tang

**Affiliations:** ^1^ Department of Oncology Surgery The First Affiliated Hospital of Xi'an Jiaotong University Xi'an Shaanxi China; ^2^ Department of Pathology Guangdong Medical University Dongguan Guangdong China; ^3^ Department of Oncology Panyu Maternal and Child Care Service Centre of Guangzhou and Hexian Memorial affiliated hospital of Southern Medical University Guangzhou China; ^4^ Department of General Surgery The First Affiliated Hospital of Xi'an Jiaotong University Xi'an Shaanxi China

**Keywords:** CD74, multidrug resistance, small cell lung cancer, YAP1

## Abstract

**Background:**

Our previous research found that YAP1 may have a role in multidrug resistance (MDR) in small cell lung cancer (SCLC). However, its underlying mechanism is unknown.

**Methods:**

In this study, we investigated the expression of YAP1 using immunohistochemical staining and assessed the relationship between the expression of YAP1 and overall survival in patients with SCLC. We established H69 stable cell lines that overexpressed constitutively active YAP1 and H446 stable cell lines that dominate negative YAP1. We conducted CCK‐8, flow cytometric analysis, and in vivo chemosensitivity experiments to evaluate the function of YAP1 in drug sensitivity apoptosis in vitro and in vivo.

**Results:**

The results indicated that patients with high YAP1 expression have shorter survival rates and more advanced disease stage than those with low YAP1 expression. YAP1 may induce MDR by inhibiting the apoptosis of SCLC. YAP1 induced MDR when YAP1 was hyperactivated, and drug sensitivity increased when YAP1 was inhibited in vitro and in vivo. CD74 was significantly correlated with YAP1 in SCLC samples. Inhibition of CD74 using ISO‐1 increased drug sensitivity significantly.

**Conclusions:**

The expression of YAP1 is significantly correlated with overall survival and disease stage in patients with SCLC. YAP1 may play an important role in these patients. We were the first to report that YAP1 can induce MDR in SCLC in vitro and in vivo. CD74 may be involved in YAP1‐induced MDR.

## Graphical abstract

The expression of YAP1 is significantly correlated with overall survival and disease stage in patients with SCLC. We were the first to report that YAP1 can induce MDR in SCLC in vitro and in vivo. CD74 may modulate the MDR of YAP1.

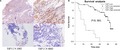

## 1 INTRODUCTION

Small cell lung cancer (SCLC), the most aggressive type of lung cancer, has a lower than 5% 5‐year survival rate and is characterized by widespread metastasis and early recurrence.^1^, ^2^ Although most patients with SCLC are sensitive to initial chemotherapy (etoposide [VP16] and cisplatin [cDDP]), many patients eventually die of rapid development of chemoresistance. The molecular mechanism involved in SCLC chemoresistance, especially multidrug resistance (MDR), remains to be fully elucidated.^3^

The main components of the mammalian hippo pathway, MST1/2, LATS1/2, YAP1, and TAZ, are evolutionarily conserved.^4^ YAP1, the transcriptional regulator of this pathway, can shuttle between the cytoplasm and nucleus. Phosphorylated YAP1 due to activated hippo pathway is sequestered in the cytoplasm.^5^ In contrast, when YAP1 translocates to the nucleus, it can induce expression of many genes related to cell apoptosis, cell growth, tumorigenesis, and metastasis.^6^, ^7^, ^8^, ^9^ In addition, YAP1 contributes to resistance to certain drugs in NSCLC.^10^, ^11^, ^12^, ^13^ YAP1 variants may be associated with the prognosis of patients with SCLC treated with platinum‐based chemotherapy.^14^ The reciprocal expression of INSM1 and YAP1 may stratify SCLC into different chemosensitivity subgroups.^15^ Our previous study found that WBP5 may induce MDR of SCLC through YAP1.^16^ However, knowledge of the role of YAP1 in SCLC is limited.

CD74 is a type II transmembrane glycoprotein initially shown to function as an MHC class II chaperone.^17^ CD74 is a multifunction protein in physiological and pathological situations and also acts as a component of the MHC class II antigen presentation pathway and cytokine receptor.^18^, ^19^, ^20^ Once it binds to the cytokine macrophage migration inhibitory factor (MIF), CD74 can induce signal transduction in many cell types.^21^, ^22^, ^23^ CD74 may be associated with cell proliferation and apoptosis in many tumor cells, including colon cancer,^24^ breast cancer,^25^ non‐SCLC,^26^ pleural mesothelioma, and melanoma.^21^, ^27^ However, little is known about the role of CD74 in SCLC.

In this study, we investigated the clinical features of YAP1 expression in SCLC patients. We analyzed the biological roles of YAP1 in vitro and in vivo and found that the function of YAP1 was significantly correlated with CD74. In conclusion, we found that YAP1 can promote MDR in SCLC and that CD74 may be associated with the regulatory mechanism of YAP1.

## 2 MATERIALS AND METHODS

### 2.1 Patients and tissue specimens

Fifty‐three patients diagnosed with SCLC after bronchofiberscopy or biopsy were identified and followed from January 2008 to March 2012 in our hospital. All patients provided informed consent and received treatment in our hospital. The Institutional Research Ethics Committee of our hospital approved the study. Clinicopathological data, including age, sex, and disease stage, are listed in Table 1.

**Table 1:** The expression of YAP1 and their relationships with the clinicopathological characteristics in SCLC patients

Variables	Total number(%)	YAP1 expression		Fisher's exact test
	n = 39	Low	High	*P* value
		n = 39	n = 14	
Age (y)				.757
≤57	26	20	6	
>57	27	19	8	
Gender				.093
Male	45	31	14	
Female	8	8	0	
Stage				.003
LD	34	30	4	
ED	19	9	10	

### 2.2 Cell culture

All cell lines in this study (H146, H446, H69, and H345) were obtained from ATCC and authenticated using STR analysis. All cells were maintained in RPMI1640 with 10% FBS and cultured in a humidified atmosphere of 5% CO_2_ and 95% air.

### 2.3 Immunohistochemical staining

Immunohistochemistry staining was performed to assess the expression of YAP1 and CD74 in tumor tissue. The specimens were dewaxed and rehydrated routinely and then soaked in 0.3% H_2_O_2_ in methanol for 30 minutes at 37°C. The sections were then incubated with anti‐YAP1 antibody (1:100; Abcam) and anti‐CD74 antibody (1:100; Abcam) overnight at 4°C. Lastly, the sections were incubated with a secondary antibody (1:500; Dako) for one hour at room temperature. Semiquantitative results were obtained, as described previously. Basically, IHC images were captured with an FSX100 microscope (Olympus), and the German semiquantitative scoring method was employed to evaluate the scores. All stained sections, including nuclei, cytoplasms, and membranes, were evaluated and scored independently by two qualified pathologists with no prior knowledge of the clinicopathological outcomes of the patients. The intensity of YAP1 staining was scored as below to quantitatively group expression levels: 0 (no staining), 1 (weak staining, faint yellow), 2 (moderate staining, light brown), and 3 (strong staining, brown). Scores >2 were regarded as high expression. Multiple simultaneous evaluations were conducted to resolve the discrepancies (<5%).

### 2.4 Quantitative reverse transcriptase polymerase chain reaction (RT‐PCR)

Total RNA was isolated from SCLC cells using the RNeasy kit (Qiagen), and cDNA was synthesized from total RNA per the manufacturer's recommendations (Tiangen).

Quantitative PCR was performed using the ABI Illumina Instrument using SYBR Green Master Mix (Tiangen). All samples were normalized to the endogenous control GAPDH, and fold changes were calculated through relative quantification (2^−△△Ct^).

### 2.5 Western blot

Equivalent amounts of protein were extracted using RIPA lysis buffer and quantified per the manufacturer's recommendations (Sigma‐Aldrich). Then, the protein lysates were electrophoresed with 10% SDS‐PAGE and transferred to a PVDF membrane. After the membrane was incubated with primary antibodies (YAP1, Abcam, 1:1000; CD74, Abcam, 1:1000), it was incubated with peroxidase‐linked secondary antibody.

### 2.6 Flow cytometric analysis

Cells were treated with different drugs (ADM, VP16, cDDP, DMSO, or Verteporfin [VP]) and collected for apoptosis, which was performed using an Annexin V/propidium iodide detection kit per the manufacturer's recommendations.

### 2.7 Colony‐forming assay

One hundred fifty cells were plated in six‐well culture plates and cultured for 14 days. Colonies were then washed with PBS three times, fixed with 4% paraformaldehyde, and stained with 0.1% crystal violet. Lastly, the colonies were counted visually.

### 2.8 Establishment of stable‐transfected cells

To generate H69 stable cell lines that overexpress constitutively active YAP1 (YAP1 with five LATS1/2 phosphorylation site mutations; YAP1‐5SA) and H446 stable cell lines that dominate negative YAP1 (YAP1‐5SA with a C terminal transactivation domain deletion; YAP1‐5SA‐△C), PEX2‐FLAG‐YAP1‐5SA or PEX2‐FLAG‐YAP1‐5SA‐△C was used per the manufacturer's recommendations. PEX2 empty vector (GenePharma) was used as the control.^28^ Stable transfections were established after selection in G418 in month one. Infection efficiency was verified by quantitative RT‐PCR and Western blot.

### 2.9 Cell viability assay

About 2 × 10^3^ cells per well were seeded in 96‐well plates and treated with different doses of drugs. After incubation with 10 μL of CCK‐8 reagent (Dojindo, Japan) for about four hours, optical density values at 450 nm were recorded. The value of cells without drug exposure was set at 100% survival. IC_50_ was calculated according to the values of cells with different concentrations of drug exposure.

### 2.10 In vivo chemosensitivity experiments

In vivo experiments were conducted as described previously.^16^ Briefly, about 5 × 10^6^ of different SCLC cells (H69‐5SA, H69‐NC, H446‐5SA‐△C, and H446‐NC) were subcutaneously injected into the flanks of nude mice. When the tumor volume reached, on average, about 150 mm^3^, the mice were treated with chemotherapeutics (ADM+cDDP+VP16). Relative tumor volume (V/V_0_) was recorded every third day.

### 2.11 Statistical analysis

All statistical analyses were done using SPSS 19.0 software. Quantitative data, presented as means ± SD, were analyzed using Student's *t* test or analysis of variance (ANOVA). Multiple comparisons were carried out using Dunnett's test. Survival curves were assessed using the Kaplan‐Meier method. Death from SCLC was the primary end point. Prognostic factors were assessed with multivariate analyses using the Cox hazards model. *P* < .05, compared with control, was considered statistically significant.

## 3 RESULTS

### 3.1 YAP1 was related to clinical stage and survival in patients with SCLC

**Figure 1:**
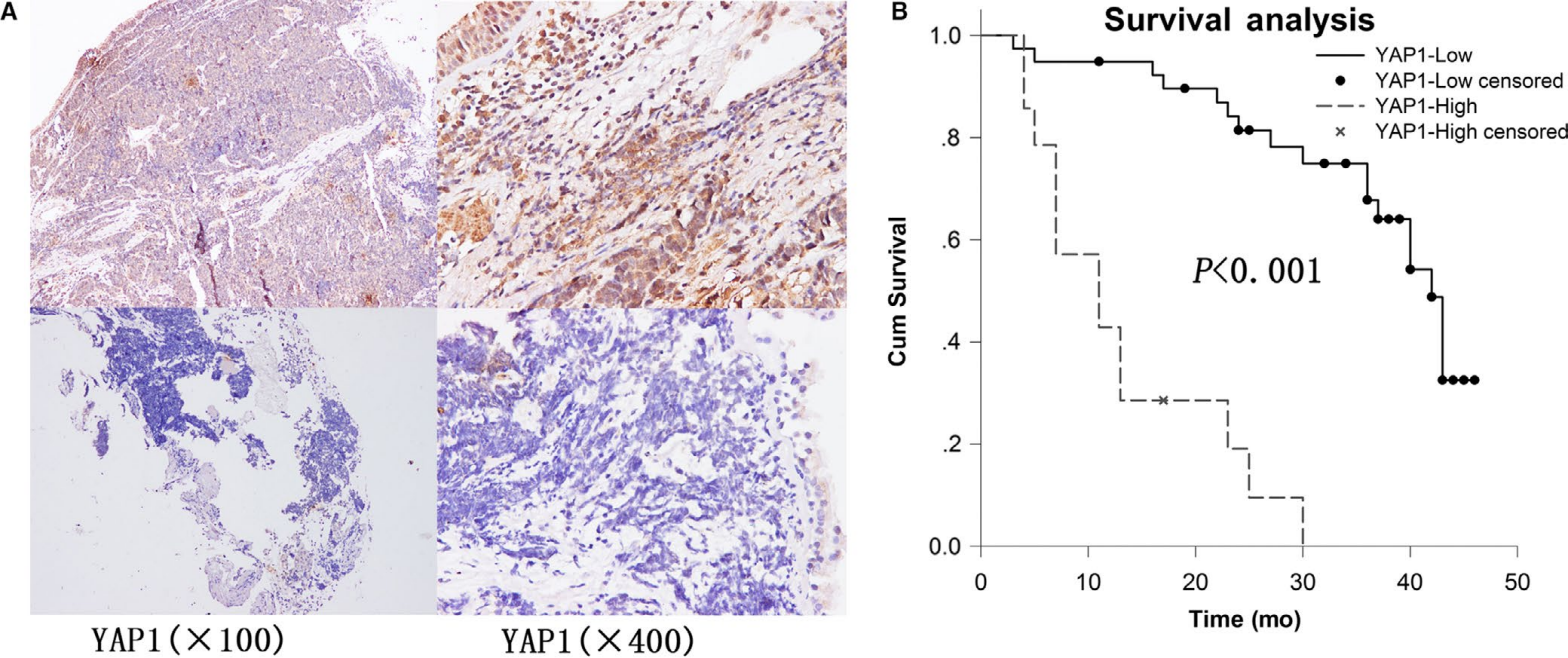
Prognostic analysis for YAP1 performed on clinical samples. A, Expression of YAP1 in small cell lung cancer (high: upper panel; low: lower panel). B, Survival differences between groups with YAP1 high and low expression assessed using the Kaplan‐Meier method

To analyze the clinicopathological features of YAP1 in patients with SCLC, immunohistochemical staining was performed in 53 SCLC samples. YAP1 was detected in both the cytoplasm and nucleus (Figure 1A). The positive rate of YAP1 was 26.42% in SCLC (Table 1). Correlation analysis showed that YAP1 was significantly correlated with disease stage (*P* = .003) but not with age or sex (Table 1). According to the results of the Kaplan‐Meier analysis, patients with high YAP1 expression had a significantly poorer survival rate than those with low YAP1 expression (*P* < .001) (Figure 1B). Cox regression analysis showed that disease stage and YAP1 expression were independent predictors of survival (Table 2). These results imply that YAP1 may indicate SCLC stage and survival.

**Table 2:** Cox regression analysis is performed using gender, stage, age and YAP1 staining age as input variables

	*P*	Exp(B)	95.0%CI for Exp(B)	
			Low	Upper
Gender	.330	0.552	0.167	1.823
Stage	.001	4.589	1.843	11.427
Age	.099	2.190	0.864	5.551
YAP1	.000	6.407	2.412	17.018

### 3.2 Manipulation of YAP1 levels in SCLC cell lines

**Figure 2:**
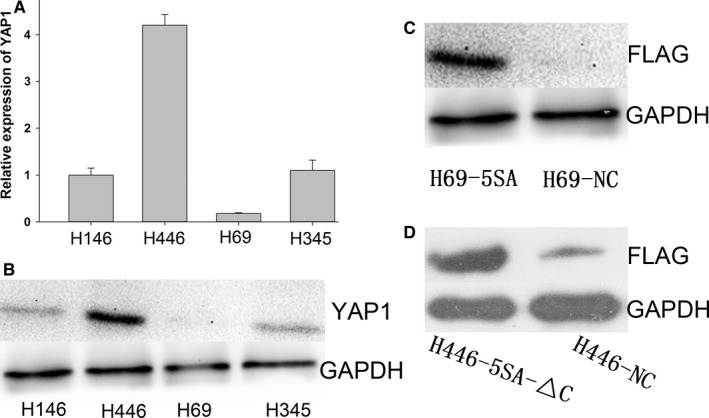
Manipulation of YAP1 levels in small cell lung cancer cell lines. A, YAP1 expression in different cell lines in mRNA and protein level (B). C, Western blot verified that H69 stable cell lines overexpressed constitutively active YAP1 (C), and H446 stable cell lines that dominate negative YAP1 (D) were transfected

YAP1 expression was measured in SCLC cell lines (H146, H446, H69, and H345) using quantitative RT‐PCR and Western blot. YAP1 expression in H446 was significantly higher than those in H146, H69, and H345, while expression in H69 was significantly lower than those in H146, H446, and H345 (Figure 2A,B).

To research the biological roles of YAP1 in SCLC, we developed H69 stable cell lines that overexpress constitutively active YAP1 and H446 stable cell lines that dominate negative YAP1, while we used H69‐NC and H446‐NC as negative controls.^28^ We then used Western blot to verify the transfection (Figure 2C,D).

### 3.3 YAP1 expression is associated with SCLC MDR, proliferation, and apoptosis in vitro

**Figure 3:**
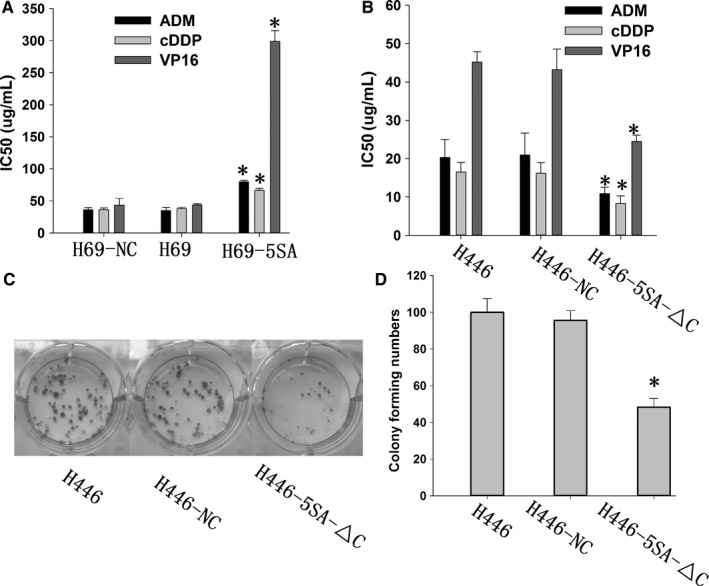
YAP1 expression is associated with small cell lung cancer multidrug resistance (MDR) and proliferation. A, Activation of YAP1 can induce MDR. B, Inhibition of YAP1 can increase drug sensitivity. C, D, Inhibition of YAP1 can inhibit proliferation

To determine whether YAP1 regulates the drug sensitivity of SCLC, we analyzed the viability of SCLC cells using CCK‐8 after exposure to different doses of drugs. We found that H69‐5SA showed markedly increased resistance to ADM, cDDP, and VP16 compared with H69‐NC or H69, while H446‐5SA‐△C showed significant sensitivity to ADM, cDDP, and VP16 compared with H446‐NC or H446 (Figure 3A,B). We also conducted a colony‐forming assay to evaluate the effect of YAP1 on cell proliferation. The results showed that the number of colonies decreased significantly in H446‐5SA‐△C compared with H446‐NC or H446 (Figure 3C,D).

**Figure 4:**
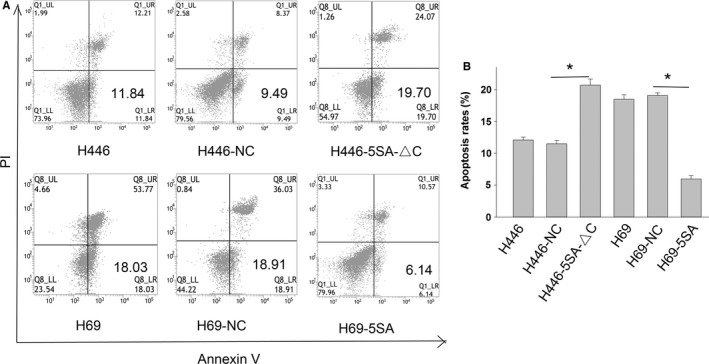
YAP1 expression is associated with small cell lung cancer (SCLC) apoptosis. Representative pictures (A) and bar chart (B) show that inhibition of YAP1 can increase the apoptosis rates of SCLC and that activation of YAP1 can decrease the apoptosis rates of SCLC when treated with ADM

We then analyzed the effects of YAP1 on cell apoptosis after drugs exposure. Apoptosis rates increased significantly in YAP1 hypoactive cells and decreased significantly in hyperactive cells compared with controls when exposed to ADM (Figure 4), cDDP (Figure S1), and VP16 (Figure S2).

**Figure 5:**
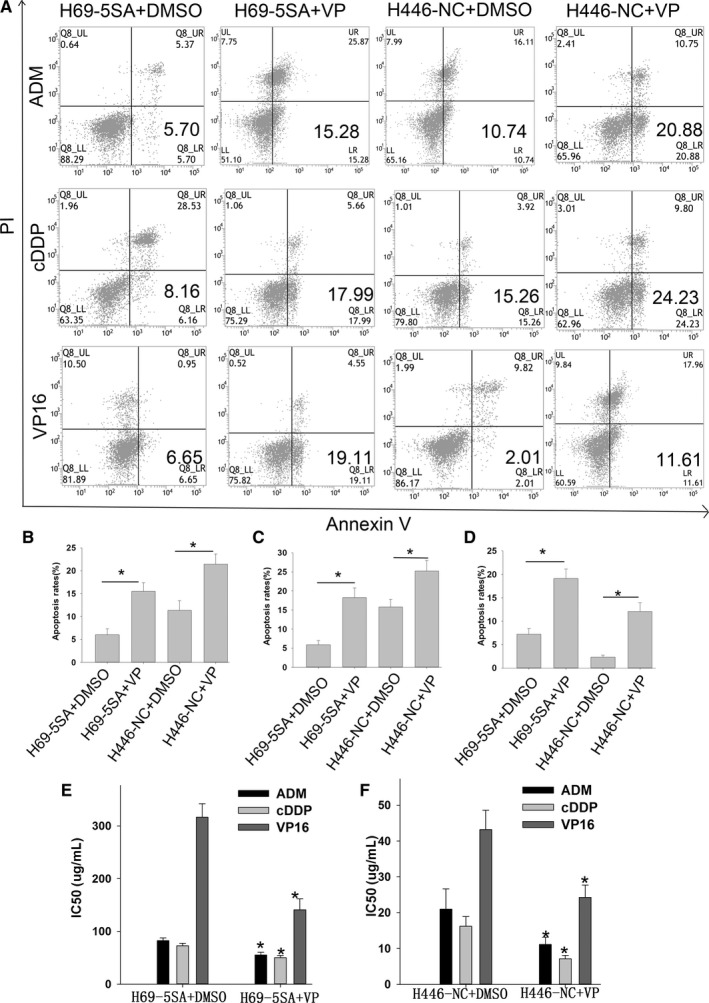
Inhibition of YAP1 by Verteporfin (VP) can increase the apoptosis rates and increase drug sensitivity in small cell lung cancer (SCLC). A, Representative pictures show that inhibition of YAP1 by VP can increase the apoptosis rate of SCLC when treated with ADM, cDDP, and VP16. Bar chart shows that inhibition of YAP1 can increase the apoptosis rate of SCLC when treated with ADM (B), cDDP(C), and VP16 (D). E and F, Bar chart shows that inhibition of YAP1 by VP can increase the drug sensitivity of SCLC

Verteporfin (VP) is a small‐molecule compound that can inhibit the activity of YAP1.^29^ To clarify the role of YAP1 in the MDR and apoptosis of SCLC, we treated SCLC cells with VP and used DMSO as a control. Inhibition of YAP1 by VP can increase the apoptosis rates of SCLC cells when treated with ADM, cDDP, and VP16 (Figure 5A‐D). Meanwhile, drug sensitivity increased significantly when VP inhibited YAP1 (Figure 5D,F). These functional experiments show that YAP1 is closely related to SCLC MDR, apoptosis, and proliferation in vitro.

### 3.4 YAP1 promotes the resistance of SCLC cells to drugs in vivo

**Figure 6:**
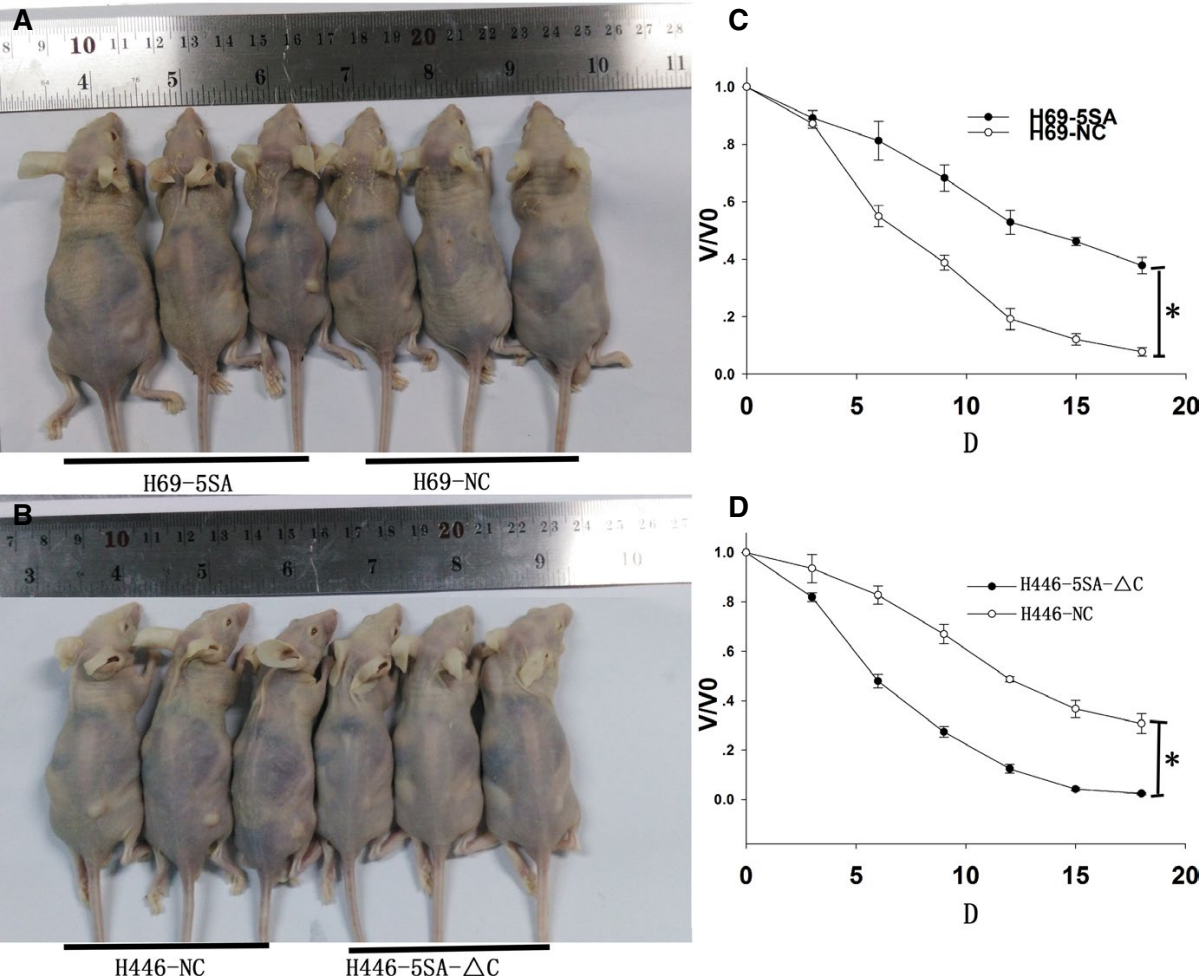
YAP1 can induce SCLC MDR in vivo. A and B, Tumor xenografts after administration of drugs. C, Activation of YAP1 can induce MDR. D, Inhibition of YAP1 can increase drug sensitivity in vivo

To investigate the role of YAP1 in the MDR of SCLC in vivo, we constructed tumor xenograft models. Four exponentially growing SCLC cells (H69‐5SA, H69‐NC, H446‐NC, and H446‐5SA‐△C) were used for in vivo chemosensitivity experiments. When tumor volume had reached, on average, about 150 mm^3^, the mice were given chemotherapeutics (ADM+cDDP+VP16).^16^ The tumor decreased more slowly in H69‐5SA and H446‐NC than in H69‐NC and H446‐5SA‐△C (Figure 6). These data suggest that YAP1 can induce SCLC MDR in vivo.

### 3.5 YAP1 promotes MDR of SCLC by CD74‐related signing pathways

**Figure 7:**
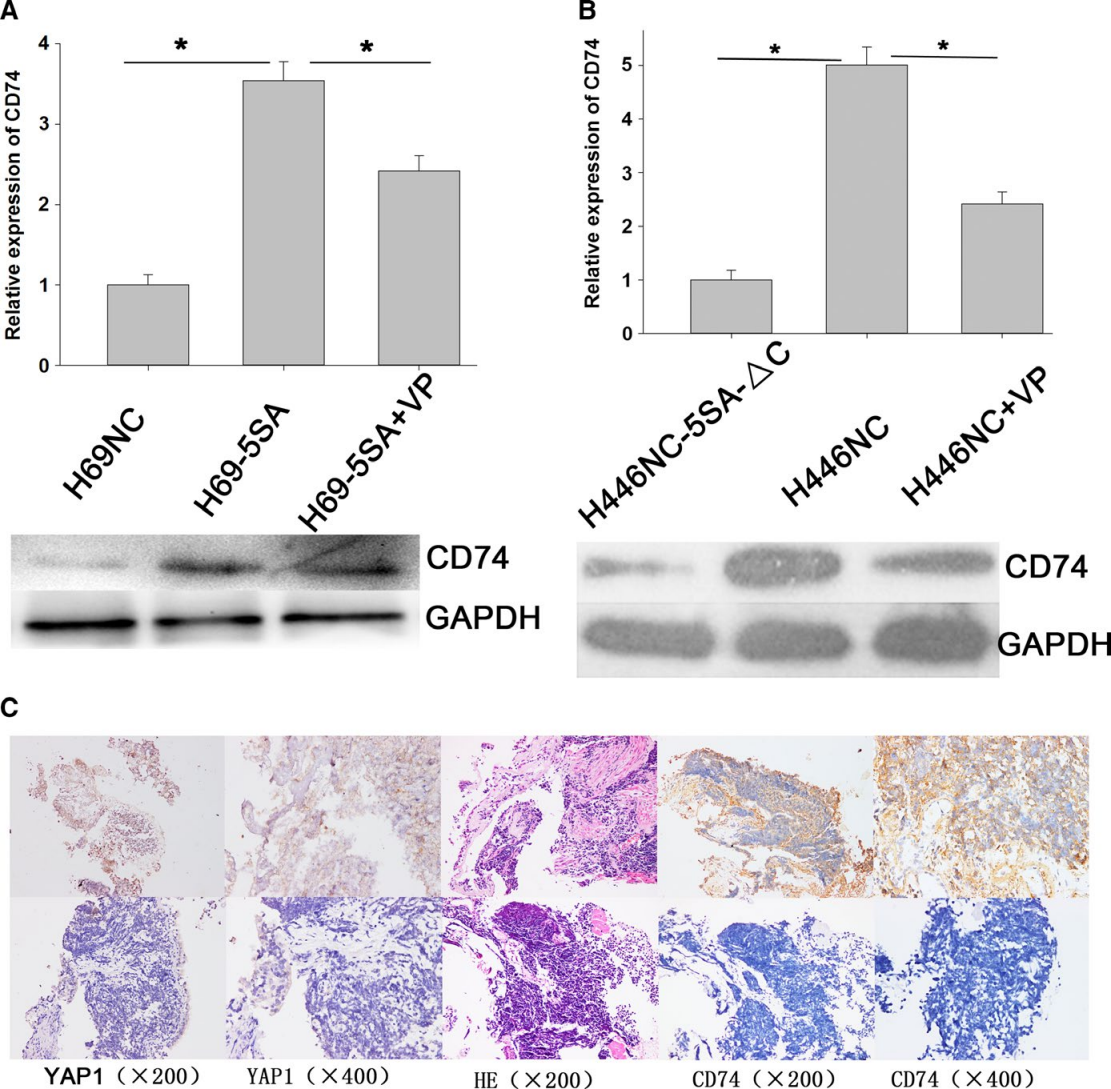
CD74 expression is significantly correlated with YAP1 in small cell lung cancer (SCLC) cells and samples. A, Quantitative reverse transcriptase polymerase chain reaction and Western blot show that activation of YAP1 can increase the expression of CD74. B, Inhibition of YAP1 can reduce the expression of CD74. C, Expression of YAP1 and CD74 in SCLC samples. Upper panels show that YAP1 and CD74 expression are both high in the same sample. Lower panels show that YAP1 and CD74 expression are both low in the same sample

**Figure 8:**
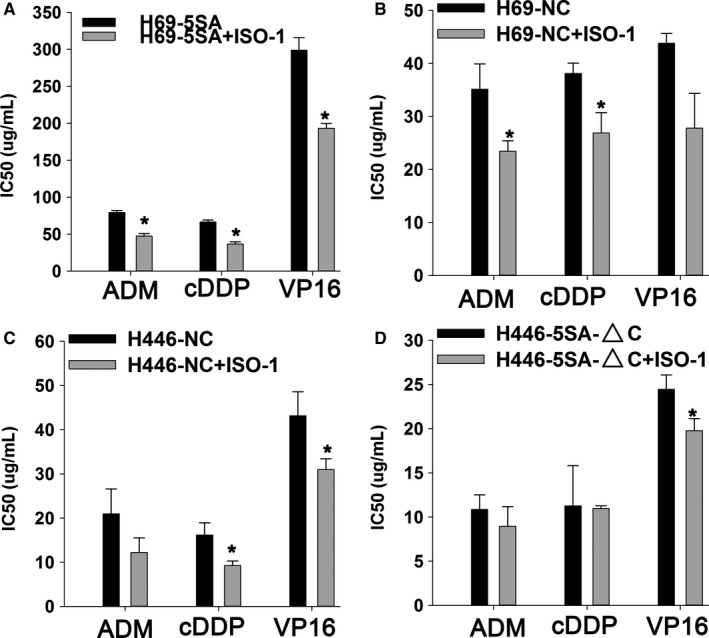
Inhibition of CD74 by ISO‐1 can increase the drug sensitivity of small cell lung cancer cells. IC_50_ decreased significantly in different cells (A: H69‐5SA; B: H69‐NC; C: H446‐NC; and D:H446‐5SA‐△C) when treated with ADM, cDDP, and VP16

CD74 expression increased significantly when YAP1 was activated, and CD74 expression decreased significantly when YAP1 was inhibited by chance (Figure 7A,B). Then, we conducted immunohistochemical staining using the same samples to explore the correlation between YAP1 and CD74 (Figure 7C). The results revealed that CD74 is significantly correlated with YAP1 in SCLC samples (Table 3). To identity the function of CD74, we used ISO‐1, which inhibits MIF binding to CD74, to inhibit CD74 activity.^21^ The IC_50_ values of CD74‐inhibited cells decreased markedly after ADM, cDDP, and VP16 treatment (Figure 8). This suggests that CD74 may be a mechanism for the effect of YAP1 on MDR in SCLC.

**Table 3:** The expression of YAP1 and their relationships with CD74 in SCLC patients

Variables	Total number(%)	YAP1 expression		Fisher's exact test
	n = 39	Low	High	*P* value
		n = 35	n = 15	
CD74 expression				0
Low	34	30	4	
High	16	5	11	

## 4 DISCUSSION

Immune therapy with Nivolumab, Ipilimumab, and Atezolizumab has shown promise in SCLC for the first time in decades.^30^ However, it may be a long time before the results of clinical trials can be widely used for SCLC treatment.^31^ The standard chemotherapy regimen still plays an important role in SCLC treatment. Hence, understanding the mechanisms of MDR is key to improving the treatment of SCLC.

YAP1 contributes to cancer development in different ways, including promoting malignant phenotypes, expanding cancer stem cells, and increasing the drug resistance of cancer cells.^32^ It was reported that high expression of nuclear YAP1 was associated with shorter survival outcome in patients with non‐small cell lung cancer (NSCLC).^33^ Silencing of YAP1 attenuates the malignant processes in NSCLC cells.^34^ However, to our knowledge, little is known about YAP1 in SCLC. In our previous study, we found that YAP1 may be involved in the MDR of SCLC.^16^ In this study, we analyzed the expression of YAP1 in 53 SCLC tissues and found that high expression of YAP1 indicates a shorter survival time and later disease stage in SCLC patients. YAP1 may be an independent prognostic factor for patients with SCLC.

To further validate the biological role of YAP1 in SCLC, we established H69 stable cell lines that overexpressed constitutively active YAP1 and H446 stable cell lines that dominate negative YAP1. Results of CCK‐8, colony‐forming, and flow cytometric analysis indicated that YAP1 can induce MDR to ADM, cDDP, and VP16 by inhibiting the apoptosis and increasing the proliferation of SCLC.

To further clarify the role of YAP1 in the MDR and apoptosis of SCLC, we treated SCLC cells with VP that can inhibit the activity of YAP1. Inhibition of YAP1 by VP can increase the apoptosis rate and drug sensitivity of SCLC cells when treated with ADM, cDDP, and VP16. These functional experiments show that YAP1 is closely related to SCLC MDR, apoptosis, and proliferation in vitro.

In addition, in vivo data revealed that YAP1 can induce MDR when YAP1 is hyperactivated and that drug sensitivity can increase when YAP1 is inhibited. Combined with the above results, it suggests that YAP1 may play an important role in the MDR, apoptosis, and proliferation of SCLC.

CD74 has been associated with tumor progression and metastasis. Its expression has been suggested as a prognostic factor in many cancers, with high expression a marker of tumor progression.^22^ However, little is known about CD74 in SCLC. Our study demonstrated that CD74 expression is highly correlated with YAP1 in SCLC cells. Immunohistochemical staining revealed that CD74 is significantly correlated with YAP1 in SCLC samples. In addition, inhibition of CD74 by ISO‐1 can increase drug sensitivity of small cell lung cancer cells significantly. Various indications indicate that CD74 may be involved in the YAP1‐induced SCLC MDR process. In order to further clarify the regulatory mechanism between CD74 and YAP1, IP experiments between the two proteins and further functional experiments including salvage experiments will be necessary.

In conclusion, our study showed that YAP1 expression is correlated with survival rate and disease stage in patients with SCLC and that YAP1 may be an independent predictive indicator in SCLC. We first reported that YAP1 can induce MDR of SCLC in vitro and in vivo. CD74 may participate in the MDR regulatory mechanism of YAP1.

## CONFLICT OF INTEREST

None.

## Data Availability

I confirm that my article contains a Data Availability Statement even if no data are available unless my article type does not require one. I confirm that I have included a citation for available data in my references section, unless my article type is exempt.
