# Supplementary figures and images for: YAP1 promotes multidrug resistance of small cell lung cancer by CD74‐related signaling pathways
Source: Cancer Med. 2019 Nov 6;9(1):259–68. doi: 10.1002/cam4.2668 (PMC6943160; doi:10.1002/cam4.2668)

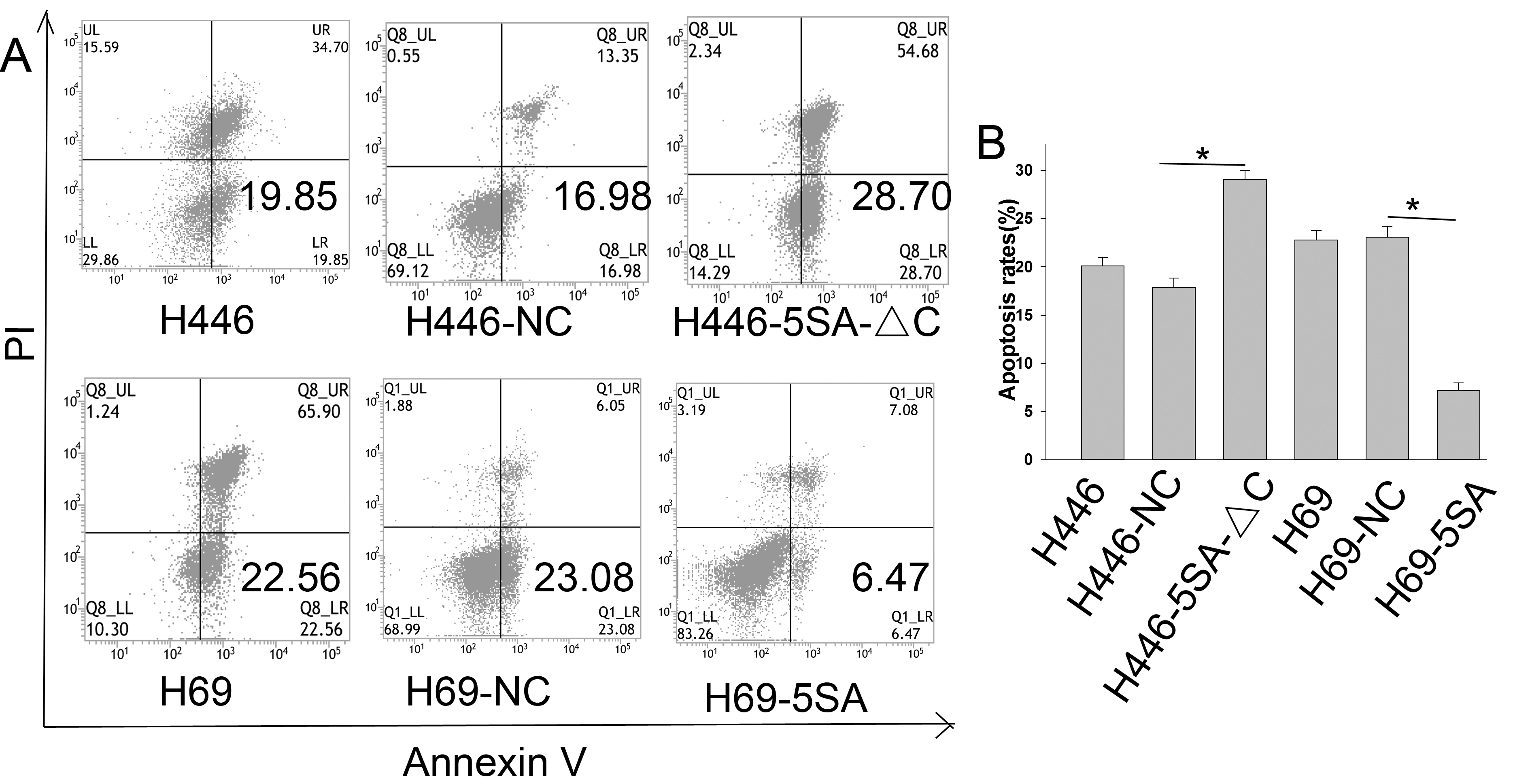

Supplement: Supplementary file 1 [file CAM4-9-259-s001.tif]

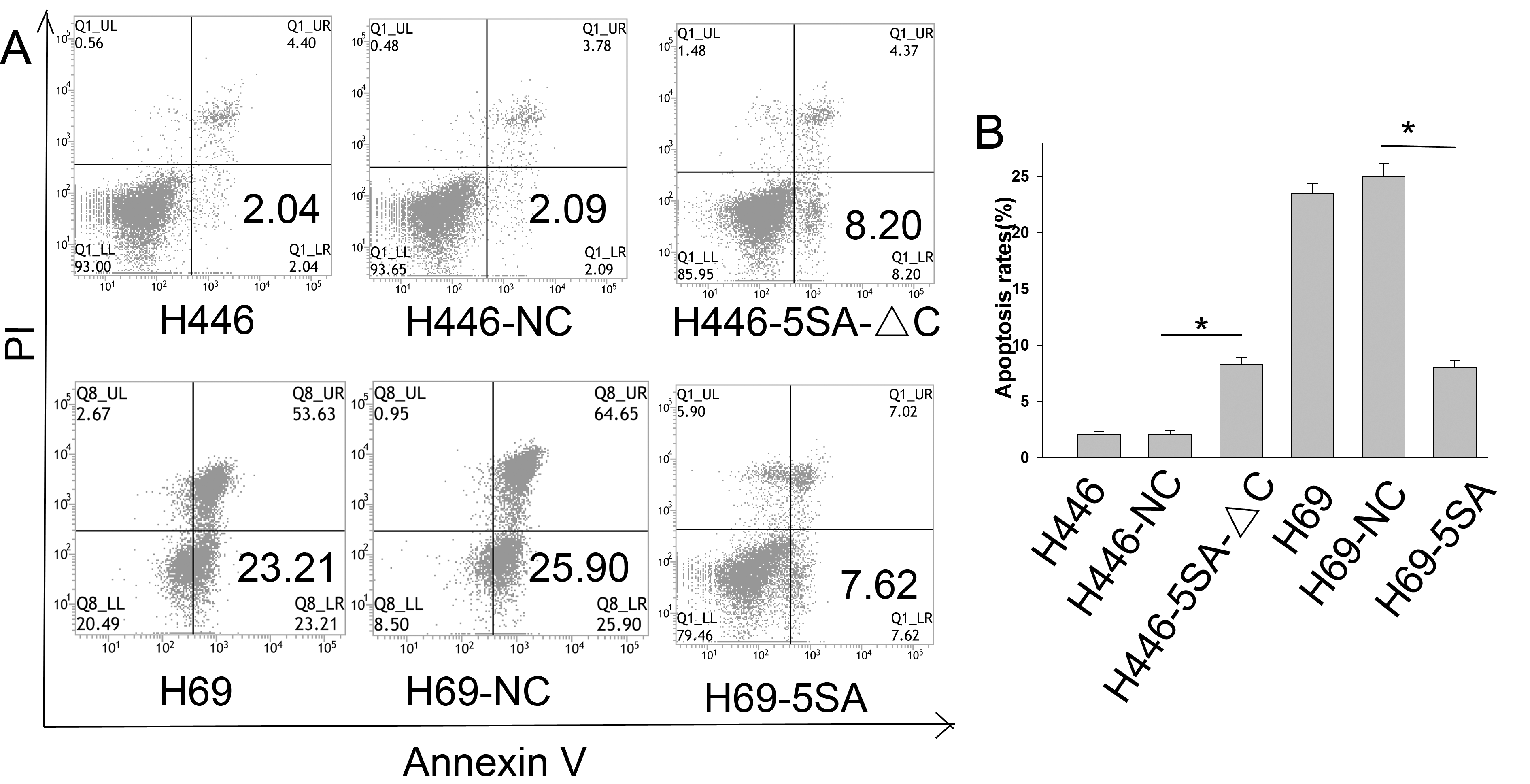

Supplement: Supplementary file 2 [file CAM4-9-259-s002.tif]
